# Expanding the clinical spectrum of cytosolic phosphoenolpyruvate carboxykinase deficiency: novel *PCK1* variants in four Arabian Gulf families

**DOI:** 10.1186/s13023-023-02946-5

**Published:** 2023-11-03

**Authors:** Marwa Al Busaidi, Feda E. Mohamed, Eiman Al-Ajmi, Nadia Al Hashmi, Khalid Al-Thihli, Amna Al Futaisi, Watfa Al Mamari, Fathiya Al-Murshedi, Fatma Al-Jasmi

**Affiliations:** 1 College of Medicine and Health Sciences, Sultan Qaboos University, Muscat, Oman; 2 Genetics and Genomics Department, College of Medicine and Health Sciences, United Arab Emirates University, P. O. Box 1555, Al Ain, United Arab Emirates; 3 ASPIRE Precision Medicine Research Institute Abu Dhabi, United Arab Emirates University, Al Ain, United Arab Emirates; 4 Department of Radiology and Molecular Imaging, Sultan Qaboos University Hospital, Muscat, Oman; 5 Department of Pediatrics, The Royal Hospital, Muscat, Oman; 6 Department of Genetic and Developmental Medicine Clinic, Sultan Qaboos University Hospital, P.O. Box 38, Muscat, Alkoudh, 123 Oman; 7 Department of Child Health, College of Medicine and Health Sciences, Sultan Qaboos University, Muscat, Oman; 8 Department of Pediatrics, Tawam Hospital, Al Ain, United Arab Emirates

**Keywords:** Phosphoenolpyruvate Carboxykinase, Hypoglycemia, Encephalopathy, *PCK1*

## Abstract

**Background:**

In metabolic stress, the cytosolic phosphoenolpyruvate carboxykinase (PEPCK-C) enzyme is involved in energy production through the gluconeogenesis pathway. PEPCK-C deficiency is a rare childhood-onset autosomal recessive metabolic disease caused by *PCK1* genetic defects. Previous studies showed a broad clinical spectrum ranging from asymptomatic to recurrent hypoglycemia with/without lactic acidosis, encephalopathy, seizures, and liver failure.

**Results:**

In this article, we discuss the occurrence of PEPCK-C deficiency in four families from the United Arab Emirates and Oman. All patients presented with unexplained hypoglycemia as a common feature. Two out of the seven patients presented with episodes of encephalopathy that resulted in seizures and neuroregression leading to global developmental delay and one patient had a neonatal presentation. Observed biochemical abnormalities include elevated lactate, transaminases, and tricarboxylic acid cycle metabolites in most patients. Elevated creatine kinase was documented in two patients. Whole exome sequencing revealed two novel (c.574T > C, and c.1268 C > T) and a previously reported splice site (c.961 + 1G > A) *PCK1* variant in the affected families.

**Conclusion:**

Patients become vulnerable during intercurrent illness; thus, prevention and prompt reversal of a catabolic state are crucial to avoid irreversible brain damage. This report will help to expand the clinical understanding of this rare disease and recommends screening for PEPCK-C deficiency in unexplained hypoglycemia.

## Introduction

If left unmanaged, low blood sugar levels during early childhood can result in permanent neurologic damage and even jeopardize the patient’s life. [1]. Normoglycemia is tightly maintained via multiple metabolic pathways such as glycogenolysis, gluconeogenesis, mitochondrial fatty acid oxidation, ketogenesis, and ketolysis [2]. Disrupting glucose homeostasis due to genetic defects in the pathways responsible for regulating glucose levels can cause hypoglycemia, which can lead to a range of disorders related to inborn errors of metabolism [3]. Disease causing variants in the *PCK1* gene (MIM 614168) lead to cytosolic phosphoenolpyruvate carboxykinase deficiency (MIM 261680). This condition is primarily characterized by the presence of fasting hypoglycemia and lactic acidosis, which can be attributed to defects in gluconeogenesis. [4]. Located on the 20q13 chromosome, the *PCK1* gene is responsible for producing the cytosolic variety of the phosphoenolpyruvate carboxykinase enzyme (PEPCK-C, EC 4.1.1.32) [5]. Like its mitochondrial isoform (PEPCK-M) expressed by the *PCK2* gene, PEPCK-C plays an important role in glucose and lipid production through their key roles in gluconeogenesis and glyceroneogenesis. Both isoforms participate in glucose metabolism through oxaloacetate conversion to phosphoenolpyruvate (PEP) occurring within distinct cellular organelles, however, their functional activities are regulated differently [6]. Unlike PEPCK-M, PEPCK-C is hormonally controlled via insulin and glucagon regulation. PEPCK-M is ubiquitously expressed while the cytosolic form is tissue-specific and mainly expressed in the liver and kidney [7, 8].

In the year 2014, a *PCK1* variant was detected in two siblings who exhibited symptoms of lactic acidosis and hypoglycemia episodes. This marked the first instance in which such a variant was clinically characterized. [9]. Various published reports have shown that individuals with PEPCK-C deficiency experience a wide range of clinical symptoms and severity levels. These can range from minimal liver involvement and normal developmental outcomes with only hypoglycemia present, to a severe and fatal disease characterized by liver failure, hyperammonemia, and encephalopathy [4, 10, 11]. To date, there are a total of 9 disease-causing variants clinically confirmed to lead to PEPCK-C deficiency in various ethnicities [12, 13]. A recent Finnish study has published an expanded clinical report on 32 PEPCK-C deficiency patients of which three adult cases were the first cases to be reported at this age [11]. This report presents first description of PEPCK-C deficiency from the Arabian Gulf region. In the four families reported in this study, two novel variants were identified in the *PCK1* gene, in addition to a previously reported variant from this population. [14].

## Materials and methods

### Patients and ethical consideration

This study was approved by Abu Dhabi Health Research and Technology Committee, reference number DOH/CVDC/2021/1318, and the College of Medicine and Health Sciences Medical Research Ethics Committee at Sultan Qaboos University, reference number SQU-EC/183/2020, MREC #2252. Affected patients were identified by the metabolic teams at Sultan Qaboos University Hospital and The Royal Hospital, Oman; Tawam Hospital, United Arab Emirates, for clinical evaluation and follow-up.

Blood samples were collected from affected patients and their parents for genetic and segregation analysis as part of their clinical examination and diagnosis. Whole exome analysis (WES) was performed by Centogene, (Germany) for Families (1, 3, and 4) and Fulgent Genetics Lab (United States) for Family 2. All participants signed an informed consent form to participate in this study.

### In-silico analysis and impact of variants on protein structure and function

*PCK1* gene transcript entry (ENST00000319441.6) and protein sequence (NM_002591.4; P35558) were retrieved from Ensembl (https://www.ensembl.org/index.html) and UniProt (https://www.uniprot.org/) databases, respectively. The effect of the filtered variants from WES on protein sequence was predicted via the ExPASy translate tool (https://web.expasy.org/translate/). To assess the effect of the detected genetic variants on protein function, different in silico prediction tools have been used. NN-Splice (http://www.fruitfly.org/seq_tools/splice.html) was used to predict the effect of the variant on splicing [15]. SIFT (Sorting Intolerant from Tolerant) algorithm (https://sift.bii.a-star.edu.sg) was utilized to predict whether the studied variants affect PEPCK-C protein function. If SIFT’s score is < 0.05, the SNP is considered tolerated; if the score is > 0.05 the SNP is considered to affect protein function [16]. PolyPhen-2 (Polymorphism Phenotyping v2) algorithm (http://genetics.bwh.harvard.edu/pph2/index.shtml) was also used to predict the impact of the detected missense variants on its structure and function using different sequence and structure-based predictive features. PolyPhen-2 scores range between 0 and 1, benign variants have scored in the range of 0 and 0.15, possibly damaging variants have a score in the range of 0.15 and 0.9 and confidently damaging variants have a score between 0.9 and 1 [17]. PremPS (https://lilab.jysw.suda.edu.cn/research/PremPS/) evaluates the effects of amino acid substitutions on protein stability by calculating the changes in unfolding Gibbs free energy [18]. The HOPE project (https://www3.cmbi.umcn.nl/hope/) has been used to analyze the structural effects of a point mutation in a protein sequence [19]. Finally, to evaluate whether these variants might be disease-causing, MutationTaster online tool has been applied (https://www.mutationtaster.org/MutationTaster69/index.html) [20]. The detected variants were initially classified according to the ACMG/AMP guideline through the Franklin by Genoox online tool (https://franklin.genoox.com). Subsequently, reclassification was conducted, taking into account the performed in silico analyses [21].

## Results

### Clinical presentation

#### Family 1.

**Fig. 1:**
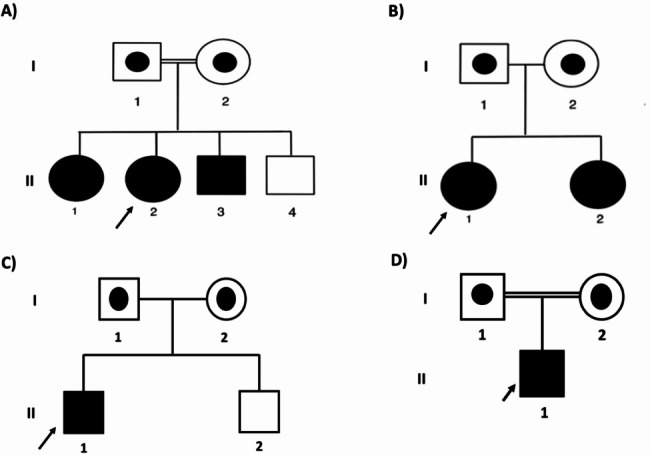
PEPCK-C deficiency affected patients’ family pedigrees. **(A)** Family 1 with three affected patients carrying the splice site c.961 + 1G > A variant **(B)** Family 2 with two affected siblings carrying the missense c.574T > C variant **(C)** Family 3 with one affected patients and **(D)** Family 4 with one affected child carrying the missense *PCK1* c.1268 C > T variant

**Fig. 2:**
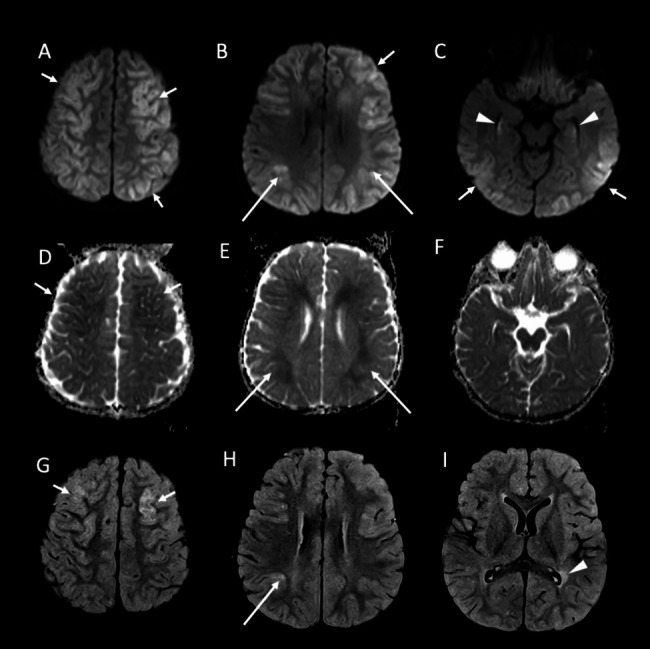
Brain MRI characteristic of PEPCK-C deficiency in the proband (Patient 1.2) of Family 1. **(A-C)** Axial diffusion-weighted images; **(D-F)** ADC map images; and **(G-I)** axial FLAIR images: There are bilateral areas of increased cortical signal intensity in diffusion-weighted images in the cerebral hemispheres (short arrows, **A-C**). Some of these areas in the superior frontal and parietal lobes show diffusion restriction (arrows in **D**) and FLAIR hyperintensity (for example, arrows in **G)**. Diffusion restriction was also present in the genu of the corpus callosum (not shown) and in the hippocampi (arrowheads, **C**). There is diffusion restriction in the deep and subcortical white matter in the frontal and parietal lobes bilaterally with focal faint FLAIR hyperintensity in the right parietal lobe (long arrows in **B, E**, and **H**). Focal peri-trigonal white matter FLAIR hyperintensities were present without diffusion restriction (arrowhead, **I**)

The proband (Table 1: 1.2, Fig. 1A: II.2) is a 10-year-old female who is the second child of a consanguineous family of Omani Arab ethnic origin (Fig. 1A). She had an unremarkable pregnancy, and birth history with normal growth and development until the age of 2.5 years when she presented with an episode of acute encephalopathy. The child appeared to be well in the morning but vomited after breakfast, became lethargic, and started to have abnormal movements. Upon her presentation to a local hospital, she had a low-grade fever and tonic-clonic seizures. There was no record of blood glucose. Physical examination revealed an encephalopathic child with a Glasgow Coma Score (GCS) of 7. She had hepatomegaly but otherwise age-appropriate growth parameters, no dysmorphic features, and no jaundice. The neurological assessment showed hypertonia and hyperreflexia. Electroencephalogram (EEG) showed epileptic encephalopathy. Magnetic resonance imaging (MRI) of the brain showed cortical T2/FLAIR hyperintensities in the cerebral hemispheres with involvement of the frontal, parietal, and occipital lobes and the posterior temporal lobes. Some of the areas of cortical signal abnormality showed diffusion restriction. In addition, diffusion restriction was present in the genu of the corpus callosum and the hippocampi. There was also diffusion restriction in the subcortical and deep white matter in the superior frontal and parietal lobes. The basal ganglia were normal (Fig. 2).

Laboratory investigations upon transfer showed elevated creatine kinase (CK) 997 U/L (Ref range: 55–170 U/L), elevated alanine aminotransferase (ALT), and aspartate aminotransferase (AST) at 687 U/L (Ref range: 7–56 U/L) and 315 U/L (Ref range: 0–35 U/L) respectively. Amino acids and acylcarnitine profiling on dried blood spots by tandem mass spectrometry were unremarkable. She was treated for acute encephalitis. Her lethargy and responsiveness had improved but she lost all her developmental milestones including head support after this episode and continued to have seizures. Eight months later, she developed another episode of lethargy and vomiting. She was found to be hypoglycemic with a blood glucose of 1.8 mmol/L (Ref range: 3.5–5.5 mmol/L), had increased anion gap metabolic acidosis, and elevated lactate of 5.5 mmol/L (Ref range: 0–2 mmol/L). She had normal ammonia, a borderline elevation of AST of 39 U/L (Ref range: 0–32 U/L), and normal CK. Urine organic acids in the second episode showed a large peak of 3-hydroxyisobutyric acid and a mild to moderate increase in glycolic, fumaric, malic, 2-ketoglutaric, adipic, octenedioic, and decenedioic acids. The echocardiogramshowed left ventricular apical trabeculation, normal cardiac function, and patent foramen ovale.

The child’s lethargy improved after the correction of hypoglycemia, but she continued to have poorly controlled seizures complicated by poor compliance with anti-epileptic medications. She was discharged home on regular carnitine supplementation at a dose of 50 mg/kg/day, recommendation to avoid fasting, and was given an emergency medical letter to initiate immediate management for hypoglycemia and metabolic acidosis in case of intercurrent illnesses. Although biochemical changes including blood glucose, lactate, transaminases, and creatine kinase became normal shortly after admission, unfortunately, the metabolic insult led to neuroregression with global developmental delay, seizures, and profound intellectual disability. She regained the ability to walk independently after a few months, but she is still unable to grasp a pencil or feed herself, became non-verbal, and is unable to respond to one-step commands. She was diagnosed with severe autism and she has developed stereotypic movements, she eats mud and stones, and she does not recognize food. Upon her last assessment at the age of nine years, she is on two anticonvulsant treatments, levetiracetam and clobazam, and still getting short episodes of generalized tonic clonic seizures twice monthly on average. MRI at the age of seven years showed interval development of diffuse cortical atrophy with prominent sulcal spaces and ex-vacuo dilatation of the lateral ventricles. Bilateral white matter T2/FLAIR hyperintensities were present due to prior insult. The last EEG at the age of eight years showed features consistent with epileptic encephalopathy. Whole exome sequencing (WES) analysis detected a homozygous splice site variant (c.961 + 1G > A) in the *PCK1* gene which was predicted to affect the transcript splicing. The variant was detected in both parents in a heterozygous form.

The proband’s elder sister 11 years old (Table 1: 1.1, Fig. 1A: II.1) is asymptomatic and has no medical concerns apart from one episode of documented hypoglycemia during severe gastroenteritis in early childhood. She is attending regular school with good performance. Targeted familial mutation analysis showed homozygous c.961 + 1G > A for the *PCK1* variant. While well, she had normal glucose, blood gas, lactate, ammonia, CK, transaminases, and plasma amino acid profile. She is currently not taking any medications but was recommended to avoid fasting and she received an emergency medical letter.

The youngest brother of the family (Table 1: 1.3, Fig. 1A: II.3) was diagnosed pre-symptomatically at the age of ten months as part of family screening. Investigations while asymptomatic at the time of diagnosis showed mild lactic acidosis 2 mmol/L (Ref range: 0.5–1.6 mmol/L) and the liver function tests showed elevated ALT and AST at 425 U/L (Ref range: 7–56 U/L) and 70 U/L (Ref range 0–35) respectively. All normalized on further repeat, but AST remained marginally elevated at 44 U/L. Parents were instructed to avoid fasting and received an emergency medical letter. At the age of three years and six months, he had an episode of gastroenteritis for two days, on the third day, he woke up with profuse sweating following which he developed status epilepticus. Upon arrival at the hospital, blood glucose was 0.7 mmol/L, metabolic acidosis was noted with a lactate of 6.9 mmol/L (Ref range 0–2 mmol/L), ammonia 33 umol/L (Ref range: 11–32 µmol/L), elevated AST 88 U/L, ALT 58 U/L and CK 518 U/L. The seizure was aborted after 4 h but the child remained encephalopathic for 24 h, responding to painful stimulation with generalized hypotonia. In addition to anticonvulsants, initial management included correction of hypoglycemia, maintenance of adequate calorie intake, supplementation of carnitine at 100 mg/kg/day, and coenzyme Q10 at 20 mg/kg/day. Although the child had fully recovered, his parents felt that he was not as alert and bright as his baseline. Currently, he is attending kindergarten with no issues at school. The formal IQ test was not done yet. The last EEG at the age of three years and nine months showed posterior bilateral delta slowing. MRI brain was normal.

#### Family 2

The proband (Table 1: 2.1, Fig. 1B: II.1) in the second family is an eleven-year-old daughter of consanguineous Omani Arab parents. (Fig. 1B). She was born at term with unremarkable perinatal and postnatal history. At the age of five years, she started to develop episodes of recurrent vomiting that were infrequent and used to occur about once per year. During the episodes, she was identified to have hypoglycemia, ketonuria, and mild lactic acidosis that were corrected by intravenous (IV) dextrose treatment. The episodes became more frequent after the age of eight years, but it was managed at home by oral high-glucose fluids. The patient remained completely well between the episodes. She has normal development and good school performance. Whole exome sequencing (WES) showed a homozygous missense (c.574T > C; p.Cys192Arg) variant in the *PCK1* gene. Although the variant was labeled as of unknown significance, several features suggest its pathogenicity as highlighted below.

The proband’s younger sister (Table 1: 2.2, Fig. 1B: II.2) is a 3 year and 5 months old child who was born at full term with unremarkable perinatal history. At four months of age, she presented with vomiting, irritability, and sweating. Her blood glucose was 1.7 mmol/L (Ref range: 3.5–5.5 mmol/L) upon arrival at the emergency room and it was corrected by intravenous dextrose treatment. The next day, she presented with vomiting again and had non-ketotic hypoglycemia (glucose: 1.5 mmol/L). Physical examination was remarkable for brisk deep tendon reflexes but was otherwise unremarkable. She had elevated lactate of 4.4 mmol/L (Ref range: 0–2mmol/L) and mildly elevated ALT and AST at 93 U/L (Ref range: 7 to 55 U/L) and 59 U/L (Ref range: 0–35 U/L) respectively. She received correction with IV dextrose and was maintained in intravenous fluids for five days until oral feeding was fully tolerated again. She was discharged home on home emergency formula and avoidance of fasting. By the age of three years, she had four additional episodes of hypoglycemia all characterized by vomiting, lethargy, and mild elevation of lactate and transaminases. Plasma amino acids showed elevated proline, alpha-alanine, citrulline, alpha-aminobutyric acid, valine, methionine, isoleucine, leucine, and lysine. Targeted variant analysis was carried out and she was identified to be homozygous for the c.574T > C (p.Cys192Arg) variant identified in her sister. Upon the last clinic evaluation at the age of three years and three months, the child had normal growth and development for age and unremarkable physical examination including no hepatomegaly. Biochemical tests including lactate, transaminases, and CK were normal.

#### Family 3

The proband is a 7-year-old male of Emirati origin who was referred to the metabolic clinic with recurrent vomiting, hypoglycemia, and metabolic acidosis (Table 1: 3.1, Fig. 1C: II.1). He was born to non-consanguineous parents, but all his grandparents are from the same tribe (Fig. 1C). His younger sibling is healthy.

The patient’s development was within normal limits. Since his early infancy, he had recurrent mild upper respiratory tract infections. At the age of 3 months, he was diagnosed with gastroesophageal reflux disease (GERD). At the age of 6 years, he was presented with asthma-like symptoms and wheezing associated with post-tussive vomiting. His vomiting episodes worsened, and the patient was admitted to the ER due to hematemesis that was a result of the Mallory-Weiss tear as the patient vomited 9 times in 6 hours. A year later, he was referred to the metabolic clinic due to metabolic acidosis (HCO_3_ 12 mEq/l) and a high anion gap of 24 mmol/l (Ref range: 8–16 mmol/L). Urine analysis was not performed. The patient reported cramp-like discomfort in his upper abdominal region, so a diagnostic sonographic assessment was done. The examination found no abnormalities or irregularities. At the age of 7 years, the patient was confirmed to have hypoglycemia and metabolic acidosis with a high anion gap. His blood tests showed normal liver function, transaminases, ammonia, lactate, CPK, and uric acid. His urine ketone levels were normal, but it was not performed during acidosis or hypoglycemia. He had normal acylcarnitine and free carnitine, but his total carnitine was slightly low. Plasma alanine was 607 µmol/l (Ref range: 300–568 µmol/l). The patient’s cardiac electrocardiogram (ECG) and echocardiogram were normal. The patient’s hypoglycemia improved with intravenous (IV) dextrose. Solo WES revealed a missense (c.1268 C > T; p.Pro423Leu) variant of unknown significance in the *PCK1* gene of the proband that was segregated from his parents and not identified in the healthy sibling as confirmed by Sanger sequencing.

#### Family 4

The proband (Table 1: 4.1, Fig. 1D: II.1) in the fourth family is a four-year-old son of a consanguineous Omani Arab parent from Sharqiyya. He was born to a G1P0 mother following an unremarkable pregnancy at full term with a vacuum-assisted vaginal delivery. The child was well after birth with no resuscitation required, and had good APGAR scores of 8 and 9 at 1 and 5 min respectively. He was appropriate for gestational age with a birth weight of 3.7 kg, length of 55 cm, and head circumference of 34 cm. He was discharged home after delivery but then he developed cyanotic spells at the age of 23 hours and appeared encephalopathic with respiratory distress and seizures. Initial investigations following collapse showed glucose of 3.4 mmol/L (Ref range: 3.5–5.5 mmol/L), Lactate of 5.9 mmol/L (Ref range: 0–2 mmol/L), ammonia of 58 µmol/L (Ref range in neonates: < 82 µmol/L), ALT 42 U/L (Ref range: 4–40 U/L), and the other liver function parameters were normal. The septic workup was negative, and head computed tomography (CT) was reported to be normal. He was discharged at the age of 23 days but presented to the emergency room at the age of 2 months with increased seizure frequency. Blood glucose was normal at 5.3 mmol/L and lactate was 2.6 mmol/L. However, his seizures eventually subsided, and he has been off anticonvulsant medications since the age of 3 years. There were no other acute episodes of encephalopathy or hypoglycemia. Assessment at the age of 3.5 years showed global developmental delay as the child was able to sit with support, respond to his name, and was babbling. Microcephaly was noted with a head circumference of -3.3 standard deviations (SD) whereas weight was on the 35th percentile and height on the 50th. On physical examination, he had lower limb hypertonia and hyperreflexia. WES revealed the same homozygous variant in the *PCK1* gene that was identified in family 3 (c.1268 C > T; p.Pro423Leu).

**Table 1:** The Demographic Data, Genotypes, and Main Clinical and Biochemical Features of the Affected Individuals

Family	Family 1			Family 2		Family 3	Family 4
**Patient**	1.1	1.2 (Proband)	1.3	2.1 (Proband)	2.2	3.1 (Proband)	4.1 (Proband)
**Age**	11 years	10 years	4 years	11 years	3 years	7 years	4 years
**Age of onset**	2 years	2.5 years	3 years	5 years	4 months	6 years	23 h
**Gender**	Female	Female	Male	Female	Female	Male	Male
**Region**	Oman			Oman		United Arab Emirates	Oman
**PCK1 variant**	c.961 + 1G > A; p.His322Glufs81* Homozygous			c.574T > C; p.Cys192Arg Homozygous		c.1268 C > T; p.Pro423Leu Homozygous	
**Main clinical features**	Asymptomatic	Fever, vomiting, encephalopathy, with lethargy, seizures, hepatomegaly, hyperreflexia, and hypertonia	Vomiting, encephalopathy, lethargy, and seizures	Recurrent vomiting	Recurrent vomiting, lethargy, and hyperreflexia	Recurrent vomiting, recurrent URTI, intermittent asthma, and hepatomegaly	Encephalopathic with respiratory distress and seizures, lower limb hypertonia, and hyperreflexia
**Main biochemical features**	Hypoglycemia was reported once during gastroenteritis illness	Hypoglycemia, elevated CK, AST, ALT, and TCA metabolites	Hypoglycemia, mildly elevated lactate, and AST	Hypoglycemia, ketonuria, and mildly elevated lactate	Hypoglycemia, mildly elevated ALT, AST, lactate, and branched-chain amino acids	Hypoglycemia Metabolic acidosis with high anion gap, and high alanine	Hypoglycemia, elevated ammonia, and lactate levels
**Outcome at last evaluation**	Normal Development	Profound intellectual disability, autism, epileptic encephalopathy	Normal development	Normal development	Normal development	Normal development	Microcephaly, and developmental delay

### Genetic analysis

The detected *PCK1* missense variants in the studied families are novel and were not reported in the human gene mutation database (HGMD) [13]. However, c.961 + 1G > A and c.1268 C > T were reported in heterozygous states in ClinVar and gnomAD databases as likely pathogenic with allelic frequencies of 0.00002459 and 0.00016, respectively. In addition, the splice site variant has been previously identified by our team as an exome reanalysis report of the proband of family 1 which was reported pathogenic as per the ACMG/AMP guidelines (Table 2) [14]. Mutation effects on protein structure and function were assessed using various in-silico prediction tools to further confirm their pathogenicity. The splice site variant (c.961 + 1G > A) identified in family 1 lies at the boundary of exon 6 and intron 5 of the gene and is predicted to abolish the donor splice site at this position as indicated via the Neural Networks splice site prediction tool (Table 2). The variant is reported disease-causing as speculated via MutationTaster at which the loss of splice site at this position will lead to the retention of intron 5 to the mature mRNA disturbing the overall reading frame. The resulting frameshift will lead to the generation of a premature stop codon at 1209 (wildtype terminates at 4320). None of the performed analyses predicted the degradation of the mutated mRNA by non-mediated decay. Therefore, the translated PEPCK-C enzyme amino acid sequence will be disturbed starting from position 322 and prematurely terminates at 403 (wildtype is 622 amino acids long). As a result, multiple critical domains and post-translational modifications will be lost or affected such as the substrate binding site at position 403–405, three GTP binding sites, and four acetylation sites (Table 2). Collectively, such changes would significantly affect the enzyme function leading to the underlying phenotypes reported in Family 1.

**Table 2:** In silico prediction analysis of c.961 + 1G > A splice site variant effect on PEPCK-C function and structure

		c.961 + 1G > A (p.His322Glufs81*)
**Exon**		6
**gnomAD** **and/or** **1000G**		rs776767788 (Total allele frequency = 0.00002459)
**Mutation taster**		Disease-causing (Leads to loss of function)
**Splice site**		Loss of the donor splice site at exon 6 / intron 5
**Effect**	**mRNA**	Frameshift due to the retention of intron 5 and the generation of a premature stop codon at position 1209
	**Protein**	Normal translation changes from residue 322 and prematurely terminates at 403
**Critical protein domains affected**		Substrate binding 403–405 GTP binding sites at 405, 436, 533 Abolished acetylation sites at 473, 521, 524, and 594
**ACMG/AMP classification**		Pathogenic (PVS1, PM2, and PP5)

The missense variants c.574T > C and c.1268 C > T detected in families 2, 3, and 4 were identified as disease-causing and damaging according to multiple pathogenicity prediction tools (Table 3). The c.574T > C variant will lead to the substitution of the neutral cysteine amino acid to the positively charged arginine at position 192 (p.Cys192Arg). Analysis of amino acid conservation indicates that the wildtype Cys192 is conserved in 59/60 mammals examined that includes 12/12 primates and 36/36 non-mammalian vertebrates increasing the likelihood that a change at this position might not be tolerated. Since the mutated residue (Arg) is positively charged, larger, and less hydrophobic compared to wildtype, amino acid modifications and interactions at this position might be affected. Additionally, such changes would overall affect the enzyme conformation and structure; thus, its functional activity. Considering the performed analyses and assessments, the underlying missense variant is collectively classified as likely pathogenic (Table 3) according to the ACMG/AMP guidelines [21].

On the other hand, substituting proline with leucine at position 423 (p.Pro423Leu) due to the c.1268 C > T missense variant may adversely affect the protein enzymatic activity due to structural malformation as predicted via the HOPE prediction tool (Table 3). The residue is located on the surface of the protein, mutation of this residue can disturb interactions with other molecules or other parts of the protein. Since Pro423 is exposed to the protein surface and close to the enzyme active site, substituting it with leucine (larger R-chain) will most likely disturb the amino acid interactions with domains critical to the enzyme active site. According to the ACMG/AMP guidelines, the underlying missense variant is categorized as likely pathogenic considering the performed in silico analyses (Table 3) [21].

**Table 3:** In-Silico Prediction Analysis of The Detected *PCK1* Missense Variants’ Effect on PEPCK-C Function and Structure

		c.574T > C (p.Cys192Arg)	c.1268 C > T (p.Pro423Leu)
**Exon**		4	8
**gnomAD** **and/or 1000G**		Not reported	rs148603002 (Total allele frequency 0.00016)
**Mutation taster**		Disease-causing	
SIFT*	**Effect**	Damaging	
	**Score**	0.01	0
**Conservation**		Highly conserved	
**PremPS**	ΔΔG** (kcal mol-1)	1.89	0.99
	**Location**	Core	Surface
**PolyPhen2†**	**Effect**	Probably damaging	
	**Score**	0.984	0.998
**Hope**		The mutant residue introduces a positive charge in a buried residue which can lead to protein folding problems. The mutation will cause a loss of hydrophobic interactions in the core of the protein.	The mutated leucine has a bulky side chain that may affect the enzyme conformation and function
**ACMG/AMP classification**		Likely pathogenic (PM2, PM3, PP3, PP4, PP5)	Likely pathogenic (PM2, PM3, PP1, PP3, PP4)

* The score ranges from 0 to 1. The amino acid substitution is predicted damaging if the score is < = 0.05, and tolerated if the score is > 0.05

** ΔΔGwt→mut (positive and negative sign corresponds to destabilizing and stabilizing mutations, respectively)

† The score ranges from 0.0 (tolerated) − 1.0 (deleterious)

## Discussion and conclusion

The PEPCK-C enzyme regulates the conversion of oxaloacetate into phosphoenolpyruvate, which is the rate-limiting step in gluconeogenesis. During the fed state, the body receives glucose from the diet and hence can get its energy requirement and store the excess amount as glycogen. However, during the fasting state, or if there was a metabolic stress in which the energy demand is very high, energy is generated through the degradation of stored glycogen to glucose (glycolysis) and via the gluconeogenesis pathway where PEPCK-C enzyme gets involved to keep blood glucose levels maintained within normal limits [22]. Therefore, the PEPCK-C enzyme is activated during periods of metabolic stress like fasting, intercurrent infections, trauma, or surgery which explains the variation between reported cases in the age of onset that is dependent on metabolic stress events and on the genetic modifiers in other stress response pathways. That may also explain the asymptomatic state while patients are well. Taking early precautions to prevent metabolic stress and control its triggers would positively impact the patients’ clinical state and eliminate the occurrence of irreversible cellular dysfunction in vital organs (i.e., brain and liver).

Most of our reported patients presented with hypoglycemia in addition to different symptoms of variable severities. The proband from the first family had vomiting, fever, lethargy, seizures, and encephalopathy accompanied by her hypoglycemia. This was followed by neuroregression, the development of epileptic encephalopathy, autism, and profound intellectual disability. Having an asymptomatic sibling (patient 1.1) and a mildly symptomatic sibling (patient 1.3) shows the variability in the disease penetrance and severity and the important role of other genetic and environmental factors in the disease manifestations. Patients from the second and third families presented with cyclic vomiting accompanied by hypoglycemia. Despite the repeated episodes of significant metabolic stress with symptomatic hypoglycemia in the affected children in families 2 and 3, the metabolic decompensation did not progress to encephalopathy or irreversible neurological dysfunction. Indeed, the biochemical findings were also milder with patients in the second and third families. Patient 2.2 from the second family showed milder elevation in her liver enzymes (ALT 93 U/L, AST 59 U/L) compared to patient 1.2 from the first family (ALT 687 U/L, AST 70 U/L). Lactate was frequently elevated during episodes of hypoglycemia but not when patients were asymptomatic reflecting the pressure on mitochondrial function during decompensation episodes. Patient 3.1 liver function test, liver enzymes, and urine ketone were normal. Patient 4.1 had neonatal onset of acute encephalopathy which eventually led to significant developmental delay and acquired microcephaly. Based on the literature review, neonatal hypoglycemia was reported in (7/32) patients with PEPCK-C deficiency but these patients did not have any documented microcephaly [10]. Seizure was observed in three of our reported patients which were most likely provoked by hypoglycemia, except for patient 4.1 who did not have any records of hypoglycemia. Vieira et al. noted that these patients have low plasma serine levels which might contribute to the acute neurological presentation but there was no measurement of serine in cerebrospinal fluid (CSF) [11], in our case there is no CSF serine measurement as well for this patient. However, it is uncertain if the neonatal metabolic decompensation in patient 4.1 explains his significant global developmental delay or if this can be explained by an additional genetic disease that was missed by WES.

Patient 1.2 also showed elevated creatine kinase of 997 U/L, which most probably reflects damage to her skeletal muscle, which is a known high-energy consumer, but CK elevation may be secondary to her status epilepticus as well. Patient 2.1 had ketonuria, although not a common finding in our cohort and previous reports and is speculated to be a hypoketotic response to extreme metabolic stress. Patients with PEPCK-C deficiency present with non/hypo ketotic hypoglycemia. This is supported by the Finnish cohort of patients with PEPCK-C deficiency who had high free fatty acids levels indicating normal activation of lipolysis and Low ketone level which is suggestive of deficient ketogenesis and the urine organic acid findings (3-hydroxyhex-4-enoic acid, 5-hydroxyhex-2-enoic acid) in those patients support insufficient ketone production [23]. Our patients shared multiple clinical and biochemical features with the previously reported patients. The main feature was unexplained hypoglycemia, which is common in most symptomatic patients. Other similar features shared between some of our patients and previous patients include lactic acidosis, TCA cycle intermediates accumulation, and elevated liver enzymes [4, 9, 24]. Unlike some of the previous reports, none of our patients had documented hyperammonemia, although ammonia was not consistently measured during all episodes.

The c.961 + 1G > A splice site variant in family 1 is predicted to disrupt the wildtype reading frame of the gene which may lead to non-mediated decay or the expression of a malformed enzyme. In this clinical report, we have identified two *PCK1* novel variants. In the second, third, and fourth families, missense variants (c.574T > C and c. 1268 C > T) were detected in a homozygous state in all affected patients. Reported missense variants are predicted to affect protein structure and critical amino acid interactions that may lead to loss of enzymatic activity and function. Measuring PEPCK-C enzymatic activities in patient samples will further confirm the genetic testing and diagnosis. It is expected that mutations affecting the protein reading frame (reported in Family 1) will lead to lower enzymatic activity. The severe splice-site mutation in the first family resulted in liability to severe symptoms and irreversible neurological injury, seen with the symptomatic patient, such as seizures and encephalopathy.

There are a total of nine genotypes reported in the literature, one splice variant, two deletion variants, and 6 missense variants. The patient with four amino acid deletion mutations in the *PCK1* gene presented with gastroenteritis, followed by unexplained liver failure, hyperammonemia, hypoglycemia, and hyperlactatemia [24]. The homozygous *PCK1* missense variant c.925G > A was reported in the largest Finnish cohort of 22 patients which showed high variability, ranging from mild to severe cases [4, 24]. The presented data in our patient showed variability in clinical presentation among the siblings (Family 1) and variability among different families with the same genotype as demonstrated in patients 3.1 and 4.1.

Affected patients become vulnerable during biochemical stress periods due to the inactive PEPCK-C enzyme and therefore prompt reversal of the catabolic state is mandatory to avoid metabolic insult to the brain, which is achievable through gluconeogenesis prevention by the avoidance of fasting and the administration of glucose polymer emergency regimen to patients under metabolic attack to eliminate irreversible outcomes [24]. Despite the variations in the reported PEPCK-C deficiency genotypes, there was a diverse array of clinical presentations observed as detected in the current and previous studies [10]. It appears that the physical characteristics of patients who have biallelic variants in the *PCK1* gene are primarily determined by environmental factors in addition to the other genetic modifiers involved in hypoglycemia and stress responses. Both external elements and genetic modifiers play a crucial role in shaping the phenotype of these individuals. Furthermore, a clear explanation of the factors that may predispose to metabolic stress to parents and the provision of home and medical emergency letters is a very important component to protect against delayed treatment and irreversible neurological insult. Since it is a treatable disease, preventing hypoglycemic recurrence could be achieved through a proper early diagnosis of such metabolic disorders. Therefore, PEPCK-C enzyme deficiency should be suspected with unexplained hypoglycemia seen in children and it has variable penetrance and severity. In a recent pilot study, Goetz et al. found elevated glutamine levels in a patient with PEPCK-C deficiency by analyzing dried blood spots collected over 55 hours [10]. PEPCK-C could be included as part of genomic Newborn screening for early diagnosis and treatment. Patients become vulnerable during biochemical stress periods; thus, prevention and prompt reversal of a catabolic state are mandatory to avoid death and a metabolic insult to the brain. Our seven patients reported will provide an expanded understanding of this ultra-rare disease.

## Data Availability

Variant data have been submitted to ClinVar with Submission IDs: SUB13310333 (https://www.ncbi.nlm.nih.gov/clinvar/variation/2502278/?oq=SUB13310333&m=NM_002591.4(PCK1):c.574T%3EC%20(p.Cys192Arg)) for c.574T > C; (p.Cys192Arg), SUB13350966 (https://www.ncbi.nlm.nih.gov/clinvar/variation/1474811/?oq=SUB13350966&m=NM_002591.4(PCK1):c.1268 C%3ET%20(p.Pro423Leu)) for c.1268 C > T; p.(Pro423Leu), and SUB13351110 (https://www.ncbi.nlm.nih.gov/clinvar/variation/931905/?oq=SUB13351110&m=NM_002591.4(PCK1):c.961%201G%3EA) for c.961 + 1G > A; (p.His322Glufs81*) variants. The data that support the findings of this study are available on request from the corresponding author.
